# Xenoestrogenic activity in blood of European and Inuit populations

**DOI:** 10.1186/1476-069X-5-12

**Published:** 2006-05-05

**Authors:** Eva C Bonefeld-Jorgensen, Philip S Hjelmborg, Thayaline S Reinert, Birgitte S Andersen, Vladimir Lesovoy, Christian H Lindh, Lars Hagmar, Aleksander Giwercman, Mogens Erlandsen, Gian-Carlo Manicardi, Marcello Spanò, Gunnar Toft, Jens Peter Bonde

**Affiliations:** 1 Unit of Cellular and Molecular Toxicology, Department of Environmental and Occupational Medicine, Institute of Public Health, Vennelyst Boulevard 6, Build. 1260, University of Aarhus, DK-8000 Aarhus, Denmark; 2 Regional Clynical Center of Urology and Nephrology, Kharkiv, Ukraine; 3 Department of Occupational and Environmental Medicine, University Hospital, SE-22185 Lund, Sweden; 4 Scanian Fertility Centre, Malmö University Hospital, SE-20502 Malmö, Sweden; 5 Department of Biostatistics, Institute of Public Health, University of Aarhus, Vennelyst Boulevard 6, Build. 1260, DK-8000 Aarhus, Denmark; 6 Laboratory of Genetics, Department of Agricultural Sciences, University of Modena and Reggio Emilia, Viale Kennedy 17, I-42100 Reggio Emilia, Italy; 7 Section of Toxicology and Biomedical Sciences, BIOTEC-MED, ENEA Casaccia, Via Anguillarese 301, I-00060 Rome, Italy; 8 Department of Occupational Medicine, Aarhus University Hospital, Noerrebrogade 44, Build.2C, DK-8000 Aarhus, Denmark

## Abstract

**Background:**

Human exposure to persistent organic pollutants (POPs) is ubiquitous and found in all individuals. Studies have documented endocrine disrupting effects and impact on reproduction. The aim of the present study was to compare the level of xenoestrogenic activity in serum of groups with varying POP exposure, and to evaluate correlations to the POP biomarkers, 2,2',4,4',5,5'-hexachlorobiphenyl (CB-153) and 1,1-dichloro-2,2-bis (*p*-chlorophenyl)-ethylene (*p,p'*-DDE).

**Methods:**

The study included 358 men: Greenlandic Inuit's, Swedish fishermen, and Warsaw (Poland) and Kharkiv (Ukraine) inhabitants. Xenoestrogenicity of serum extracts alone (XER) and XER competitive (XERcomp) effect on 17β-estradiol induced estrogen receptor (ER) transactivity were assessed in the hormone free, lipophilic serum fraction containing the POPs using the MVLN human breast cancer cell line.

**Results:**

No agonistic XER activity was exhibited for Inuit serum samples, while 12 – 24% of the European samples had detectable agonistic XER activity. On the contrary, 71% of Inuit serum samples antagonized XERcomp compared to 7 – 30 % in the other regions. XER and XERcomp were not or weakly correlated to the two POP markers. XER activity of Inuit samples was negatively associated to levels of CB-153 and *p,p*'-DDE. For the Warsaw group a positive and negative correlation between XER and *p,p'*-DDE and estradiol equivalence level and CB-153 levels was found.

**Conclusion:**

No strong consistent association between xenoestrogenic net activity and the two POP markers was found. The results showed that the selected POP markers alone can not predict the integrated xenoestrogenic serum activity. Correlations to the POP markers were found at the extreme edge; the Inuit's and Warsaw study groups eliciting high frequency of samples with ER antagonistic and agonistic activity, respectively. We suggest that the variation in xenoestrogenic serum activity reflects differences in POP exposure mixture, genetic factors and/or life style factors.

## 1. Introduction

Human exposure to environmental contaminants is ubiquitous and affects also individuals living far away from the source of contaminants such as industries, combustion and waste disposal sites. Mainly caused by dietary exposure and due to their resistance to environmental and biotic degradation persistent organochlorine pollutants (POPs), e.g. chlorinated pesticides and polychlorinated biphenyls (PCBs), accumulate in body fat tissues of humans and animals. Many POPs can mimic hormone activities and are, therefore, potential endocrine disrupters (EDs) suspected to increase the risk of cancer, birth defects, reproductive and neuro-immune disorders [1-3]. To date, no clear-cut evidence for adverse endocrine-related human health effects of POPs have been obtained at the individual or population level. However, data from studies on wild-life species, laboratory animals and biomarker effects *in vitro *have strengthened the need for further research to address the concern.

The sex steroid receptors such as the estrogen receptor α (ERα) and ERβ and the androgen receptor (AR) belongs to the nuclear receptor family and are generally ligand-dependent transcription factors [4-6]. Their genomic mediated pathway include steps as binding of ligand to receptor, translocation into nucleus and binding of the receptor-ligand complex to a specific DNA response element causing a gene response. Androgens and estrogens are not male or female hormones only. Both hormones are important in both sexes and an absolute necessity for reproductive development including male fertility [7]. The steroid androgen hormones, testosterone and dihydrotestosterone, are necessary for the normal male phenotype and the presence of ERα and ERβ in human foetal testis and epididymis cells indicates that estrogens play a physiological role in regulations of spermatogenesis in mammals [8-10].

There is extensive data of *in vitro *mechanism based models on endocrine-related POPs effects on sex hormonal systems [11-14]. Although much less potent compared to 17β-estradiol (E2) the three PCB congeners CB-138, CB-153 and CB-180, comprising up to 50% of the bio-accumulated sum of PCBs, elicit a receptor mediated antiestrogen and/or antiandrogen *in vitro *activity [15], and similarly some hydroxylated PCBs exert low potency estrogenic and/or antiestrogenic effects [16,17]. Generally PCBs do not bind to the sex hormone binding globulin (SHBG) or at very low affinity [18], which may cause a higher bio-availability of the compounds. Animal studies have indicated that prenatal exposure to POPs as PCBs, dioxins and *p,p'*-DDE is associated with reduced male fertility [19-24], whereas the effect of POPs on human fertility is still controversial [25,26], and therefore an integrated risk assessment of EDs is needed [27]. *In vitro *studies of xenoestrogen mixtures present at or below their no-observed-effect concentration (NOEC) or sub-NOEC was demonstrated to cause a dramatic additive enhancement of hormone actions [28,29]. Rather than monitoring the level of identified xenoestrogens, the integrated biological activity was used to estimate the xenobiotic burden of serum samples using [17,30-32].

The present study was a part of the EU supported research project Inuendo [33]. The specific aim of the present study was to compare xenoestrogenic activities across Inuit's and European regions, and to evaluate whether the serum levels of the POP biomarkers, CB-153 and *p,p*'-DDE, were associated to the actual xenoestrogenic net activity in blood.

## 2. Subjects and methods

### 2.1 Study population and collection of blood samples

Based on earlier reports of POP body burdens four populations were included in the Inuendo study [33] for a cross-sectional fertility study representing a wide range of POP burdens. For the present study the aim was to recruit 300 male spouses to pregnant women in Greenland, Warsaw (Poland) and Kharkiv (Ukraine), and a subgroup (n = 100) of an already established cohort of fishermen from Sweden was also included in the study [25]. A general criterion for eligibility was that the participants had to be born in the country of study and at least 18 years of age. Serum samples were randomly selected from the main Inuendo study groups, and the intension was to analyse serum samples including high and low POP exposure within each study group. However, because of the limit in project timing and in some cases too little blood was collected for the analysis, serum samples were randomly selected before POP determination, and fewer serum samples were analyzed. Males were recruited during 2002–2004 in Greenland (n = 72; Sisimiut, West Coast n = 50, and Tasiilaq, East Coast n = 22), in Warsaw, Poland (n = 99) and in Kharkiv, Ukraine (n = 88) [25]. Earlier studies have reported high POP levels in Greenlandic Inuit's and differences in levels between habitants of the East and West Coast of Greenland [34,35]. However to keep similar power in the four study groups the statistical evaluation was carried out on the combined Greenlandic data only.

Information about demographic and lifestyle factors as age, body mass index (BMI), intake of seafood, coffee, smoking habits, and alcohol consumption was collected by interviews (Table 1) [25,35]. Venous blood samples were collected into 10 ml vacuum tubes and after centrifugation the serum was transferred to Nunc tubes and stored at -80°C for later analysis [35]. The study was approved by the local ethical committees representing all participating populations and all subjects signed an informed consent.

**Table 1:** Demographic and life style characteristics of men in the study groups

		***Greenland n = 72***	***Warsaw n = 98***	***Sweden n = 100***	***Kharkiv n = 88***	***All n = 358***
		
**Age**	median	30 (70)	30 (97)	47 (96)	25 (85)	31 (348)
years	*min-max*	*18–46*	*24–46*	*24–68*	*16–45*	*16–68*
						
**BMI**	median	26 (72)	25 (96)	26 (99)	24 (86)	25 (353)
Kg/m^2^	*min-max*	*12–38*	*19–38*	*22–37*	*19–36*	*12–38*
						
**Alcohol**	median	2.0 (36)	3.5 (86)	n.a	2.5 (66)	3.0 (188)
drink/week	*min-max*	*0–21*	*0–30*		*0.2–15*	*0–30*
						
**Smoking **ever	%	89 (71)	50 (97)	60 (98)	82 (87)	68 (353)
						
**Seafood**	median	1.5 (68)	1.0 (89)	n.a	4.0 (86)	2.0 (243)
days/week	*min-max*	*0–9.0*	*0–9.0*		*1.0–9.0*	*0–9.0*
						
**Coffee**	median	3.0 (62)	2.0 (83)	n.a	2.0 (37)	2.0 (182)
cups/day	*min-max*	*0–20*	*0–6.0*		*0.5–7.0*	*0–20*
						
**Total testosterone**	median	15 (36)	13 (76)	12 (99)	18 (88)	14 (299)
nmol/l	*min-max*	*3.2–25*	*6.5–23*	*4.2–28*	*8.4–32*	*3.2–32*
						
**Estradiol**	median	59 (35)	71 (76)	67 (99)	78 (88)	70 (298)
nmol/l	*min-max*	*31–89*	*45–297*	*25–154*	*33–144*	*25–297*

The values in the bracket are the numbers of individuals with data for the specific demographic and life style characteristics.

### 2.2 Determination of CB-153 and p,p'-DDE in serum

Serum concentrations of CB-153 and *p,p'*-DDE were determined using solid phase extraction (SPE) and on-column degradation of lipids followed by analysis with gas chromatography mass spectrometry as described [35]. CB-153 and *p,p'-*DDE levels were adjusted for serum lipids. Levels of detection, coefficients of variation and participation in quality control programs have been described in detail elsewhere [35,36].

### 2.3 SPE-HPLC fractionation of the serum samples

To obtain the serum fraction containing the actual mixture of bio-accumulated POPs a solid phase extraction (SPE) followed by high-performance liquid chromatography (HPLC) fractionation was performed on 3.6 ml serum [37]. Similar to the described methods [17,30,38] the POPs were extracted from the serum by SPE separation on a Spherisorb Si 60 analytical column (250 × 4.6 mm i.d., 5 μm particle size, Waters, Milford, MA, USA). This is a normal phase column and thus the separation is based on lipophilicity. This crude serum extract was then further processed using HPLC in order to separate the POPs from the endogenous hormones. The first fraction (F1: 0.00–5.30 min, protected from light in brown tubes) was set up to include most POPs while leaving out endogenous hormones by testing 18 lipophilic chemicals including PCBs, pesticides, akylphenols, bisphenol A, 6 endogenous hormones (estrogens and androgens), and 1 oral contraceptive on the HPLC system using retension times as discriminating factor. This F1 SPE-HPLC fraction was evaporated to near dryness and frozen for later ER mediated chemical activated luciferase gene expression (ER-CALUX) analysis. ER-CALUX analyses of the HPLC fractions F1, F2.1 (5.30 – 12.0 min) and F2.2 (12.00 – 14.5 min) verified that the endogenous hormones were separated from F1 but found in F2.1 and F2.2. Moreover, comparison of SPE-HPLC F1 extracts obtained by our system with F1 extracts obtained from another laboratory (Institute of Public Health, University of Southern Denmark) elicited same responses in the ER-CALUX analyses [37].

Two pools of blood bank serum (Aarhus Sygehus, Denmark), one male (KHM) and one female (KHF), were distributed into tubes with 3.6 ml and stored at -80°C. One sample from each sex was weekly processed by the SPE-HPLC method in parallel with the project samples serving as serum controls for the cleanup procedure. The day to day inter assay coefficient of variation (CV) of the SPE-HPLC + ER-CALUX analyses of these control blood samples were ≤ 13%.

#### 2.3.1 Dissolving the SPE-HPLC samples

The SPE-HPLC extracts samples (project samples and controls) were thawed and protected from light during handling. Sample solvent, 20 μl EtOH:H_2_O:DMSO (50:40:10), was added to each sample tube and the samples were placed in an Eppendorf Thermomixer Comfort at 550 rpm and 37°C for 15 minutes. Then 200 μl growth media [0.5% Dextran-Charcoal treated foetal calf serum (DC-FCS, Hyclone, Bie & Berntsen, Aarhus, DK) in phenol red-free Dulbecco's Modified Eagle's Medium (DMEM) (Gibco, Invitrogen, Taastrup, DK) supplemented with 4 mM glutamine (Sigma-Aldrich, Vallensbaek Strand, DK) and 1.28 mg/ml garamycin (Schering-Plough, Dassel, Germany)] was added to each sample and the shaking at 550 rpm and 37°C was continued for another 15 minutes. Then each sample was transferred to two new test tubes (100 μl/tube) each containing 400 μl growth media with or without the normal ER ligand E2, respectively, and used for ER-CALUX determinations.

### 2.4 ER-CALUX assay

The stable transfected MVLN human breast cancer cell line (kindly provided by M. Pons, France) carrying the endogenous ERα and ERβ genes, and the introduced estrogen-response-element (ERE)-luciferase reporter vector [39] was used for determination of ER-CALUX activities as described [40]. The measured luciferase data was corrected to cell protein (internal control), and expressed in relative light units per microgram protein (RLUs/μg protein). All controls and SPE-HPLC F1 serum extracts (100 μl/well) were analysed in triplicate. If one of the triplicate values deviated more than 30% the mean was calculated from two wells only. An E2 dose-response control in the concentration range from 0.05 pM to 500 pM (solvent 96% ethanol = 0.1%) was performed in parallel each analysis day as described [14,40]. The maximal effective concentration (EC_100_), the half maximum effective concentration (EC_50_), and EC_40 _of E2 was calculated to 150 pM, 33 pM and 25 pM, respectively, by fitting the dose-response data into Chapman, 4 parameter sigmoid equation curve using Sigma Plot 8.0 (SPSS, Chicago, IL, USA). The E2-EC_100 _and the E2-EC_40 _were used as parallel positive controls on each plate in each assay. Xenoestrogenic activity (XER) was determined by exposure of the cells to serum extracts alone and XERcomp activity upon co-exposure of the cells to serum extract + 25 pM E2 (EC_40_). The solvent controls of XER and XERcomp activity were sample solvent only and the sample solvent plus 25 pM E2 (EC_40_), respectively (see 2.3.1 for handling procedure), and for XER and XERcomp activity calculation the respective solvent controls (RLU/μg protein) were set to 1. Finally, the data was given as activity per ml serum and the values of the solvent controls were 3.13 RLU/ml serum.

The E2 equivalence (XER-EEQ) value of the agonistic XER activities was obtained by interpolation of data onto the Chapman 4 parameter sigmoid curve using the dose-response E2 Sigma Plot curve. Both the average ER-CALUX inter-CV of the solvent controls and intra-individual CV for serum sample extracts were below 5%.

No cell toxicity on MVLN determined by CellTiter 96 Cell Proliferation assay from Promega (Madison WI, US) [40] was observed after exposure to SPE-HPLC extracts.

### 2.5 Statistical analysis

The comparisons of means between XER, XERcomp and XER-EEQ were performed by the Oneway-ANOVA test. When ANOVA indicated significant group difference complementary multiple comparison *ad hoc *tests were used. The test for homogeneity of variance was performed with Levene's test. The least significant difference (LSD) pair wise multiple comparison test was used for the variables with equal variance (p ≥ 0.05) and Dunnett's T3 test was used for the variables with an unequal variance (p ≤ 0.05).

The association in each study group between POP marker and xenoestrogen activity was evaluated by means of Spearman's rank correlation. The overall association between the POP marker and xenoestrogen activity across the study groups (combined data) were evaluated by comparing the regression lines for each study group (multiple regression analysis).

Our hypothesis is that a potential determinant of POP bioaccumulation might also be a potential determinant for serum xenoestrogenic activity. As known from the literatures and also from an assessment of the total Inuendo study populations [25,35] age and seafood are such determinants. Moreover, lifestyle characteristics (Table 1) were evaluated as potential determinants of XER and XERcomp levels of combined data and the separate study groups. Using multivariate linear regression analyses, assessing the impact of the POP biomarkers on XER and XERcomp, the impact of potential confounders were evaluated by entering blocks of variables together with either CB-153 or *p,p'-*DDE as follows: In the first step age and seafood intake (continuous variables) were included in the model, and in the second step additionally smoking status (smoked ever yes/no), BMI, and coffee intake was included as continuous variables. Alcohol consumption was only recorded for limited number (n = 188, see Table 1) of subject and not for the Swedish study group. Therefore, the second step was carried out with and without alcohol consumption in the potential confounder model, which did not change the overall pattern of non-adjusted to adjusted data. Due to many missing values on the potential confounders (Table 1) the number of available observations in the confounder analyses are much smaller than in the unadjusted analysis on the full dataset (full dataset (XER, XERcomp and POP markers) N = 348; first step of confounders: N = 231; second step: N = 172). A reduction of the number of observations with 50% might introduce serious selection problems, and hence the confounder analyses might lack greater validity.

The XER and XERcomp activities were determined in protein free serum extracts free of endogenous estrogens and testosterone. As a method verification Spearman's rank correlation analyses of XER and XERcomp against determined blood levels of estradiol and testosterone (total and free) [41] were performed on the combined study group data.

Normal distribution was assessed by Q-Q plots. To improve normality and homogeneity of variance, the XER and XERcomp activities as well as lipid adjusted CB-153 (lip-CB-153) and *p,p'*-DDE (lipDDE) were natural logarithmic transformed and the statistical analyses were performed on the ln-transformed data. The POP variables were treated as continuous variables. The statistical analysis was performed in SPSS 10.0 (SPSS Inc, Chicago, IL) with the significance level p ≤ 0.05.

## 3. Results

### 3.1 Basic characteristics, CB-153, *p,p*'-DDE and xenoestrogenic serum levels

The distribution of demographic and lifestyle factors (Table 1) and serum CB-153 and *p,p'*-DDE median levels (Table 2) that may potentially influence the ER-mediated activities of the 358 adult males in this study were similar with that obtained for the total Inuendo study population [25,35]. Correlation between serum concentration of CB-153 and *p,p'*-DDE was found with higher correlations in Greenland (r_s _= 0.94) and Sweden (r_s _= 0.75), while relatively lower correlation was observed for the study group of Kharkiv (r_s _= 0.45) and Warsaw (r_s _= 0.28).

**Table 2:** Xenoestrogenic serum activities, estradiol equivalents and lipid adjusted CB-153 and *p,p'*-DDE in serum of the study groups

		**Greenland**	**Warsaw**	**Sweden**	**Kharkiv**	ALL*^4^	All study group data*^5^
XER*^1^**RLU/ml serum**	N	72	98	100	88	358	0.003
	**Median**	**2.89**	**3.09**	**3.04**	**3.15**	**3.05**	
	**Mean**	**2.92**	**3.30**	**3.22**	**3.17**	**3.17**	
	*Min*	*1.0*	*2.4*	*2.4*	*1.0*	*1.1*	
	*Max*	*6.0*	*6.5*	*12*	*8.0*	*12*	
	*% agonist*	*1*	*21*	*12*	*14*	*-*	
	*% antagonist*	*35*	*5*	*12*	*17*	*-*	
XER-EEQ pg/g lipid*^2^	N	1^♣^	21	10	11	43	0.63
	**Median**	-	**103**	**76♥**	**139**	**114**	
	**Mean**	-	**166**	**161**	**179**	**171**	
	*Min*	-	*44*	*50*	*80*	*44 *	
	*Max*	-	*516*	*364*	*580*	*580*	
XER comp*^3^**RLU/ml serum**	N	72	94	94	88	348	< 0.001
	**Median**	**2.65**	**2.96**	**2.90**	**2.88**	**2.86**	
	**Mean**	**2.69**	**3.29**	**2.89**	**2.87**	**2.95**	
	*Min*	*2.0*	*1.8*	*1.0*	*1.1*	*1.0*	
	*Max*	*3.8*	*7.0*	*6.8*	*4.5*	*7.0*	
	*% add/syn*	*1*	*13*	*3*	*1*	*-*	
	*% antagonist*	*71*	*7*	*19*	*30*	*-*	
**CB-153 ng/g lipid**	N	74	100	98	82	354	< 0.001
	**Median**	**220**	**16**	**210**	**47**	**79**	
	*Min*	*5.1*	*33*	*4.1*	*5.5*	*3.3*	
	*Max*	*5500*	*130*	*1500*	*200*	*5500*	
**DDE ng/g lipid**	N	74	100	98	82	354	< 0.001
	**Median**	**630**	**570**	**240**	**880**	**560**	
	***Min***	***66***	***240***	***55***	***324***	***55***	
	***Max***	***13000***	***2100***	***2300***	***12000***	***13000***	

In the independent assays significant differences between the triple serum extract determinations and their respective solvent controls (% agonists/additive/synergistic) and % antagonists, were tested by the Student's t-test (Microsoft Office Excel, significance level p = 0.05). *^1^: XER: Xenoestrogenic agonistic or antagonistic activity of serum extract alone: the solvent control = 3.13 RLU/ml serum. % agonistic and % antagonistic indicates the % of samples eliciting a significantly increase or decrease in XER activity compared to solvent control, which was set to 1. *^2^: XER-estradiol equivalence (XER-EEQ) of the samples eliciting significantly agonistic activity; data calculated by interpolation to the E2 dose response curve using the sigma plot program. ♥ : One Swedish sample was too high to calculate the XER-EEQ. ♣ : One serum sample from Greenland only had an agonistic action, thus no XER-EEQ value is given. *^3^: XERcomp; XER competitive activity of serum extract + 25 pM 17β-estradiol (E2-EC_40_); % add/syn: additive/synergistic and % antagonistic indicates the % of samples responding with a further increase or a decrease of the E2-EC_40 _induced activity, respectively, compared to the E2-EC_40 _solvent control = 3.13 RLU/ml serum, which was set to 1;. *^4^**: **XER activity includes 358 samples, however, only 348 of theses samples were applied to the XERcomp analyses. All of the samples had CB-153 and *p,p'*-DDE determined. *^5^: One-way ANOVA (p value) evaluation of the combined study group data except for XER-EEQ including only the European groups. In transformed data was used.

**Figure 1:**
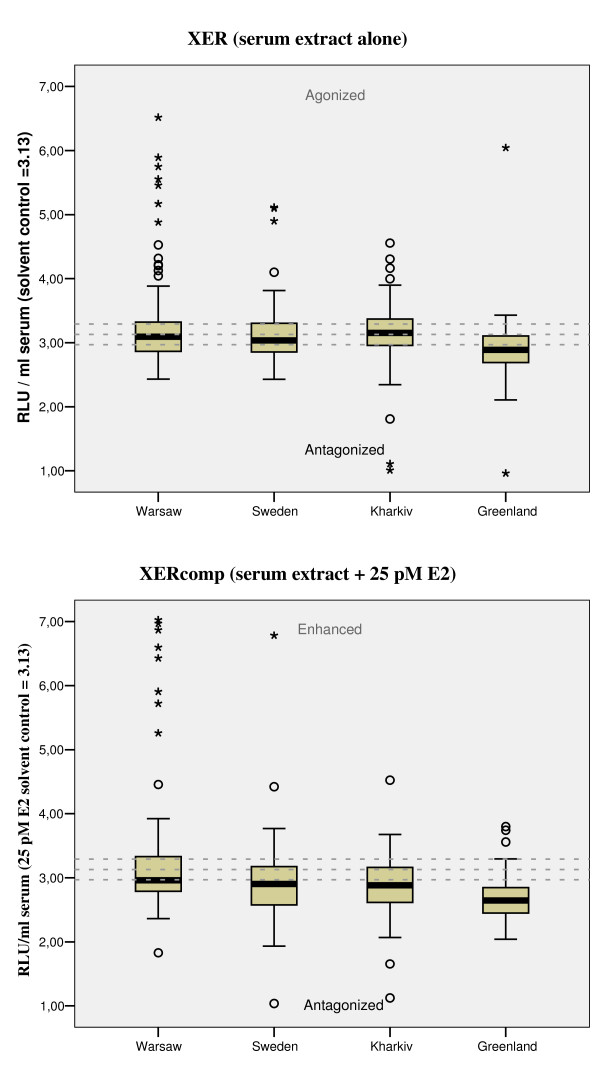
**The xenoestrogenic CALUX activity of study groups**. (A) Agonistic activity of serum extracts alone (XER) and (B) competitive XER activity upon coexposure with 25 pM E2 (EC_40_) (median, quartiles (25% and 75%) and extreme variables). For the Swedish study group an extreme agonistic (A) RLU value of 12.02 was determined (not shown). The reference line of the respective solvent controls ± SD (3.13 ± 0.16) are given as dotted lines.

The Greenlandic serum extracts elicited predominantly antagonistic effect on XER (35%) and XERcomp (71%), whereas the European samples elicited relatively higher frequency of XER agonistic activity (12–21%) of which the Warsaw samples had the highest mean of XER/XERcomp (Table 2 and Figure 1).

Oneway ANOVA analyses showed significant differences between the study groups (Table 2). Multiple comparisons of means showed that Inuit XER activities significantly differed from each of the three European study groups (p ≤ 0.03); whereas the XER activity and XER-EEQ of the European study groups did not mutually differ. Only one sample from Greenland elicited XER agonistic activity and thus no XER-EEQ data was given for the Inuit's. The XERcomp activity of the Warsaw group differed from all other study groups (p ≤ 0.004).

### 3.2 Associations between xenoestrogenic activity and CB-153 and *p,p*'-DDE

XER activity for Inuit's showed an inverse correlation to the *p,p'-*DDE levels (r_s _= -0.29; p = 0.02) and borderline inverse to CB-153 (r_s _= -0.22; p = 0.07). For the Warsaw study group the XER activity showed a significant positive correlation to the *p,p'*-DDE data (r_s _= 0.21; p = 0.04), and for XER-EEQ a significant negative correlation to CB-153 (r_s _= -0.45; p = 0.04) was found (See Additional file 1: Spearman's correlation analyses between xenoestrogenic serum activities and the level of CB-153 and *p,p'*-DDE). No further correlations between XER, XERcomp or XER-EEQ and any of the two POP markers were found. Adjustment for the potential confounders in the multivariate regression model did not give any different results for the impact of the POP biomarkers on XER and XERcomp as compared with the unadjusted models, neither for the combined data or each study group (data not shown).

### 3.3 Multiple regression of xenoestrogenic activity on POP data across the study groups

**Figure 2:**
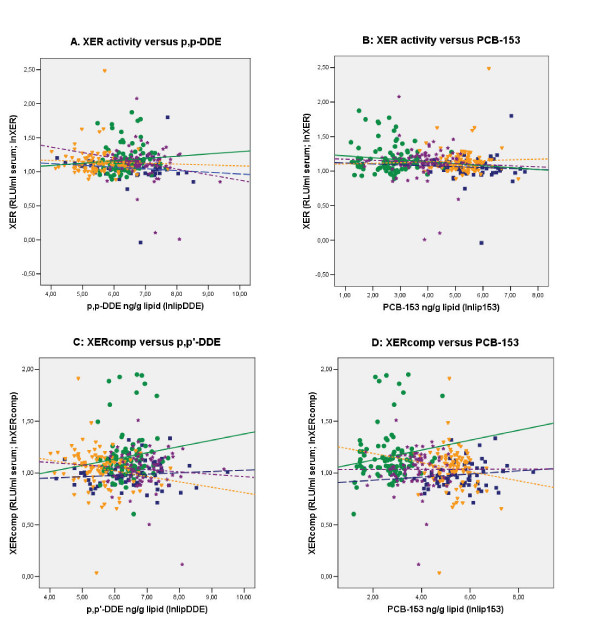
**Schematic illustration of xenoestrogenic activities related to the POP markers**. Xenoestrogenic (XER) and competitive XER (XERcomp) serum activities related to CB-153 and *p,p'*-DDE levels for the four country based study groups as a relation between (A) XER and *p,p'*-DDE, (B) XER and CB-153, (C) XERcomp and *p,p'*-DDE, and (d) XERcomp and CB-153. The values are given as ln transformed data.

Scatter plots of XER or XERcomp against CB-153 or *p,p'*-DDE for the four study groups are shown in Figure 2. Multiple regression analysis of both response variables (XER and XERcomp) showed homogeneity of slopes between study groups and CB-153/*p,p'*-DDE (Table 3), i.e. parallel regression lines among study groups. Furthermore, a model with parallel regression lines showed significant differences between the intercepts of the study groups (Table 3). Thus the differences in XER/XERcomp between study groups found by Oneway ANOVA (Table 2) and multiple comparisons still exist after adjustment for CB-153 or *p,p'*-DDE. Finally, we note that although correlations was observed between XERs and the POP markers for the Greenlandic and Warsaw study groups (Supplement), for the combined data the associations between XER/XERcomp and CB-153/DDE, as measured by the common slopes, are very weak and not statistically significant (Table 3).

**Table 3:** Multiple regressions of the combined study group data

**Response variable**	**Exposure variable**	**Homogeneity of slopes (p-value)**	**Common slope Estimate (SE) p-value**	**Common intercept (p-value)**	**Adjusted R- square**
**XER (N = 348)**	CB-153	0.86	-0.01 (0.01), 0.34	0.05	0.029
	*p,p'*- DDE	0.24	-0.03 (0.02), 0.12	0.005	0.034
**XERcomp (N = 338)**	CB-153	0.20	0.01 (0.02), 0.45	<0.001	0.087
	*p,p'*- DDE	0.15	-0.006 (0.02), 0.71	<0.001	0.086

Response and exposure variables are ln-transformed in the analysis. Homogeneity of slopes: p-value for the test for homogeneity of slopes between study group and exposure variable. Common slope: the estimated common slope across study groups assuming no interaction. Common intercept: p-value for the test of a common intercept across study groups assuming a common slope. Adjusted R-square assuming a common slope.

### 3.4 Correlations between xenohormone activities and endogenous hormone levels

To verify the exclusion of endogenous hormones from the SPE-HPLC- F1 serum extracts used for CALUX activity measurements, the XER, XER-EEQ and XERcomp results were evaluated for possible correlation to blood estradiol (pmol/L) and testosterone (nmol/L) (free and total) levels. No correlations were found between the serum XER/XERcomp activities and blood sex hormone levels neither for the combined data nor for the separate study groups.

## 4. Discussion

In the present study we determined the actual xenoestrogenic net activity in serum of adult men from Greenland, Sweden, Warsaw (Poland) and Kharkiv (Ukraine), representing populations with different POP exposure patterns (Table 2) [34,35,42,43]. For the combined data no strong consistent correlations between serum xenoestrogenic activity and the two POP proxy markers, CB-153 and *p,p'*-DDE, was observed. Significant correlations between marker POPs and xenoestrogenic serum activity was found for Inuit's and the Warsaw group, having the lowest and highest ratio of agonist:antagonist samples, respectively; an inverse association of XER activity to the *p,p'*-DDE and CB-153 levels for Inuit's, and for the Warsaw group a positive and a negative association of XER to *p,p'*-DDE and XER-EEQ to CB-153, respectively. Because of the high inter-correlation between CB-153 and *p,p'-*DDE for the Inuit's it can not be assessed which of the two POPs exert the main impact. However, for the Warsaw group the very low concentration of CB-153 and low inter-correlation of marker POPs indicates that *p,p'*-DDE might have a positive effect on XER activity, and CB-153 an inverse impact on agonistic XER as elicited by XER-EEQ. Overall, these data might be explained by the high level of PCBs in Greenland with predominant anti-estrogenic actions [15,17,44-46] and relatively high level of *p,p'*-DDE in the Warsaw group having weak estrogenic action [17,28,47,48]. However, that does not explain why no correlations were observed for the Swedish and Kharkiv groups since they had a CB-153 and *p,p'*-DDE marker profile similar to Greenland and Warsaw, respectively. Multiple regression analyses showed, that the determined differences of XER and XERcomp activity between the study groups could not be explained by the two selected POP proxy markers alone neither in combination with the measured potential confounders in the present study. However, the combined XER vs. CB-153 data showed homogeneity across the study groups, had common slopes and similar intercept (p = 0.05, Table 3, Figure 2), and assuming a common intercept a significant (β = -0.132, p = 0.01) negative correlation between CB-153 and XER activity was found. Future studies might elucidate whether our observations are serendipitous.

Between the groups the XER and XERcomp activities differed significantly. The Inuit's clearly differed from the European study groups eliciting no agonistic but high frequency of samples (71%) with antagonistic action. In contrast, the European serum samples exerted both agonistic (12 – 21% of the samples) and antagonistic effects (7 – 30% of the samples).

The heterogeneous pattern of xenoestrogenic correlations to CB-153/*p,p'*-DDE levels and also the differences in xenoestrogenic activity among the study groups might be explained by different exposure profiles e.g. : i) the estrogenic effects elicited by ER transactivity is a consequence of the combined net effects of many POPs having either ER agonistic or ER antagonistic actions; ii) the two selected POP markers do not reflect and/or represent the chemicals primarily responsible for the determined xenoestrogenic effects; iii) the combined response of the serum mixture is significant affected although the concentration of the single compounds found in human serum is lower than that needed to elicit a response of the compound on its own in an *in vitro *system; and finally iv) the net XER/XERcomp activities is the net response of exposure patterns, genetic background and life style factors. In summary, based on the two selected POP markers only, it is hard to predict the net xenoestrogenic impact *in vivo *as well as in the present *ex vivo *xenoestrogenic measurements.

The use of CB-153 and *p,p*'-DDE in serum as index biomarkers of POP exposure has been supported by several previous studies [49-52]. However, it must be taken into consideration that the relative concentrations of non-coplanar and co-planar PCBs can differ between regions depending on varying exposure sources. The ratio between serum levels of CB-153 (and other non-coplanar congeners) and co-planar PCBs was higher in Canadian Inuit's than in Canadian Caucasians from the Arctic area [53], which calls for caution using CB-153 as a global exposure marker for POP as well as for xenoestrogenic serum activities. In the present study it might be expected that the ratio between non-dioxin-like, non-coplanar PCBs (e.g. CB-153) and co-planar PCB (e.g. CB-126) also is higher for the Greenlandic Inuit's compared to the three European study groups.

*In vitro *studies demonstrated that the combined effect of several xenoestrogens including POPs at sub-NOEC exerted an additive effect, which led to dramatic change in estrogenic *in vitro *activity [28,29]. To elucidate potential human and wildlife responses to the combined impact of accumulated xenoestrogens it remains to be demonstrated mechanistically how chemicals with antiestrogen effects can modify the response of chemicals acting estrogenic in a concerted action. It can be argued that xenoestrogenic equivalents contribute only a few percent to the endogenous hormone activity – even when possible higher bioavailability of the xenobiotics is taken into consideration. However, considering the further increase of XERcomp above the reference control (25 pM E2) we calculated (using Sigma Plot and the data of the E2 dose-response curve) for this subgroup of men (n = 86) a further mean activity increase of 47% and 21% in relation to the minimum (34 pM) and mean (70 pM) endogenous estradiol level, respectively. Since the level of endogenous estrogens is much lower in male than in female the xenoestrogenic activity might have higher impact on health risk in males.

In studies using similar approach as the present no correlation between neither CB-153 nor *p,p'*-DDE and xenoestrogenicity were found. In contrast to our determination of the more specific impact of serum xenoestrogens on ER transactivation, the other studies used the E-screen MCF-7 cell proliferation as end point [17,30-32]. Moreover, we analyzed the xenoestrogenic action in male blood samples whereas the other four studies were focused on female adipose tissues [30-32] and female serum from pregnant and non-pregnant women [17]. These studies determined a higher frequency (~ 60–70%) of subjects with significantly increased estrogenic activity in the non-polar serum fraction of Spanish and Faroese women. For non-pregnant Danish women the frequency of subjects exceeding the background level was 22.7%, similarly to the frequency determined for the European males in the present study. In support to our data it was recently reported that high levels of PCBs in Slovakia male serum samples were associated with a decreased ER mediated activity and increased AhR mediated activity [46]. Recently, estrogenicity of adipose tissue extracts including bio-accumulated xenoestrogens was shown associated with higher risk of breast cancer in leaner women [32].

### 4.1 Conclusions and perspectives

Across the four study groups no strong consistent correlation between xenoestrogenicity and the two POP proxy markers was found. Thus, the CB-153 and *p,p'*-DDE alone are not optimal global POP markers of the integrated xenoestrogenic serum activity. However, between the study groups a clear difference in serum xenoestrogenicity as well as differences in correlation to the CB-153 and *p,p'*-DDE proxy markers was found. Correlations to the POP markers were found for the two study groups at the extreme edge: Inuit's with high sample frequency of antiestrogenicity, and the Warsaw group with higher frequency of samples with estrogenicity. In support to POP biomarker determinations, the present *ex vivo *biomarker xeno-activity assay may be useful in assessment of geographical surveys for the impact of environmental xenoestrogens, reflecting differences in POP exposure patterns.

On the other hand, exposure assessment based upon measurements of the actual xenoestrogenic serum activity of the isolated SPE-HPLC, F1- POP- fraction may fail to detect effects accomplished 1) by other non-lipophilic compounds in serum, 2) through other receptors or 3) by other mechanisms. More work still needs to be done to fully understand to which extent *ex vivo *measurements of receptor-transactivation reflect the *in vivo *situation.

## Competing interests

The author(s) declare that they have no competing interests.

## Authors' contributions

ECB-J drafted the work and was the main responsible for design, performance and data evaluation of the specific project; PHJ, TSR and BSA performed the mechanistic work; CHL performed POP determinations in blood; ME was the main responsible for the statistical work; JPB, AG and LH designed the overall Inuendo project. JPB and GT coordinated the execution of the project and GT had main responsibility for creating the joint database. All authors participated in the design of the study and commented on the final manuscript.

## Supplementary Material

Additional File 1 **Title: Spearman's correlation analyses between xenoestrogenic serum activities and the level of CB-153 and *p,p'*-DDE**. ln-transformed and POP lipid adjusted data was used. For definition of XER, XERcomp and XER-EEQ see legend to Table 2. Statistical significant data is given in bold. ^1^: Spearman's inter-correlation between CB-153 and *p,p'*-DDE, respectively. Click here for file
