# Supplementary material for: Xenoestrogenic activity in blood of European and Inuit populations
Source: Environ Health. 2006 May 5;5:12. doi: 10.1186/1476-069X-5-12 (PMC1481538; doi:10.1186/1476-069X-5-12)
Supplement: Additional File 1 — Title: Spearman's correlation analyses between xenoestrogenic serum activities and the level of CB-153 and p,p'-DDE. ln-transformed and POP lipid adjusted data was used. For definition of XER, XERcomp and XER-EEQ see legend to Table 2. Statistical significant data is given in bold. 1: Spearman's inter-correlation between CB-153 and p,p'-DDE, respectively. [file 1476-069X-5-12-S1.pdf]

Additional file 1: Spearman's correlation analyses between xenoestrogenic serum activities and the level of CB153 and *p,p'*-DDE.

	Greenland			Warsaw			Sweden			Kharkiv		
	n	r_s	p	*n*	r_s	p	*n*	r_s	p	*n*	r_s	p
**CB153**												
XER	*70*	-.22	.07	*98*	.02	.89	*98*	.007	.95	*82*	.13	.26
XER-EEQ*	-	-	-	*21*	**-.45**	**.04**	*10*	.07	.86	*11*	-.15	.67
XERcomp	*68*	.15	.21	*94*	.07	.50	*92*	-.13	.21	*82*	.03	.81
***p,p'*-DDE**												
XER	*70*	**-.29**	**.02**	*98*	**.21**	**.04**	*98*	-.04	.72	*82*	-.09	.42
XER-EEQ*	-	-	-	*21*	-.13	.57	*10*	.12	.75	*11*	-.09	.79
XERcomp	*68*	.11	.37	*94*	.13	.21	*92*	-.18	.08	*82*	.02	.85
CB153/ DDE[1]	*70*	**.94**	**<.001**	*98*	**.28**	**<.01**	*98*	**.75**	**<.001**	*82*	**.45**	**<.001**

ln-transformed and POP lipid adjusted data was used. For definition of XER, XERcomp and XER-EEQ see legend to Table 2. Statistical significant data is given in bold. [1]: Spearmans inter-correlation between CB-153 and *p,p'*-DDE, respectively
